# Dynamics of action potential firing in electrically connected striatal fast-spiking interneurons

**DOI:** 10.3389/fncel.2013.00209

**Published:** 2013-11-14

**Authors:** Giovanni Russo, Thierry R. Nieus, Silvia Maggi, Stefano Taverna

**Affiliations:** Department of Neuroscience and Brain Technologies, Istituto Italiano di TecnologiaGenoa, Italy

**Keywords:** fast-spiking interneurons, striatum, gap junctions, GABA, action potential

## Abstract

Fast-spiking interneurons (FSIs) play a central role in organizing the output of striatal neural circuits, yet functional interactions between these cells are still largely unknown. Here we investigated the interplay of action potential (AP) firing between electrically connected pairs of identified FSIs in mouse striatal slices. In addition to a loose coordination of firing activity mediated by membrane potential coupling, gap junctions (GJ) induced a frequency-dependent inhibition of spike discharge in coupled cells. At relatively low firing rates (2–20 Hz), some APs were tightly synchronized whereas others were inhibited. However, burst firing at intermediate frequencies (25–60 Hz) mostly induced spike inhibition, while at frequencies >50–60 Hz FSI pairs tended to synchronize. Spike silencing occurred even in the absence of GABAergic synapses or persisted after a complete block of GABA_A_ receptors. Pharmacological suppression of presynaptic spike afterhyperpolarization (AHP) caused postsynaptic spikelets to become more prone to trigger spikes at near-threshold potentials, leading to a mostly synchronous firing activity. The complex pattern of functional coordination mediated by GJ endows FSIs with peculiar dynamic properties that may be critical in controlling striatal-dependent behavior.

## Introduction

Parvalbumin-expressing, fast-spiking interneurons (FSIs) represent a small fraction of the total cell population in the striatum (~1%; Luk and Sadikot, 2001), yet they provide a key contribution to sensorimotor integration and functional coordination of striatal network activity (Parthasarathy and Graybiel, 1997; Gage et al., 2010; Tepper et al., 2010; Berke, 2011). FSIs project a feed-forward inhibitory input onto striatal principal cells, the medium spiny neurons (MSNs) (Pennartz and Kitai, 1991; Koos and Tepper, 1999; Gustafson et al., 2006; Mallet et al., 2006; Taverna et al., 2007; Gittis et al., 2010; Planert et al., 2010), and are connected to each other via electrical and GABAergic synapses (Kita et al., 1990; Fukuda, 2009). Several studies demonstrated that gap junctions (GJ), either alone or in coordination with GABAergic synapses, mediate a tight synchronization of FSI spiking and entrainment in gamma-band coherent activity in non-striatal brain areas (Gibson et al., 1999; Tamas et al., 2000; Hormuzdi et al., 2001; Szabadics et al., 2001; Traub et al., 2001; Bartos et al., 2002). In the striatum, FSIs are known to (i) fire repetitive bursts of action potentials (APs) at an intra-burst frequency of ~40–50 Hz (Bracci et al., 2003; Plotkin et al., 2005; Taverna et al., 2007) and (ii) express functional GJ (Koos and Tepper, 1999). Recent *in vivo* studies, however, showed that FSIs fire mostly asynchronously during striatal-dependent behavioral tasks (Berke, 2008; Gage et al., 2010) and in certain conditions may exert a direct inhibitory effect onto each other (Lansink et al., 2010). A recent modeling paper suggested that striatal GJ mediate a shunting effect which reduces spike firing in FSI pairs receiving uncorrelated excitatory inputs (Hjorth et al., 2009). Moreover, no significant synchronization was detected in simulated FSI striatal networks connected by both GABAergic synapses and GJ (Humphries et al., 2009). These findings suggest that synaptic connectivity between striatal FSI may be characterized by distinctive properties which promote fast inhibition rather than synchronous firing. Yet, it is unknown how GJ and GABAergic synapses shape the firing activity in live striatal FSIs, therefore a direct assessment of functional communication between these cells is needed. Here, we sought to identify the role played by electrical and GABAergic synapses in coordinating the firing activity of FSI pairs using dual patch-clamp recordings in mouse neostriatal slices. We found that GJ promoted fast, frequency-dependent inhibition of postsynaptic firing along with a slower membrane potential coupling effect which induced a loose firing coherence between simultaneously recorded cells. AP inhibition was induced by the action of the slow hyperpolarizing component of postsynaptic spikelets. Such effects were correlated with the strength of GJ conductance (Gc) and were independent of the presence of GABAergic synapses.

## Materials and methods

### Electrophysiology

**Figure 1:**
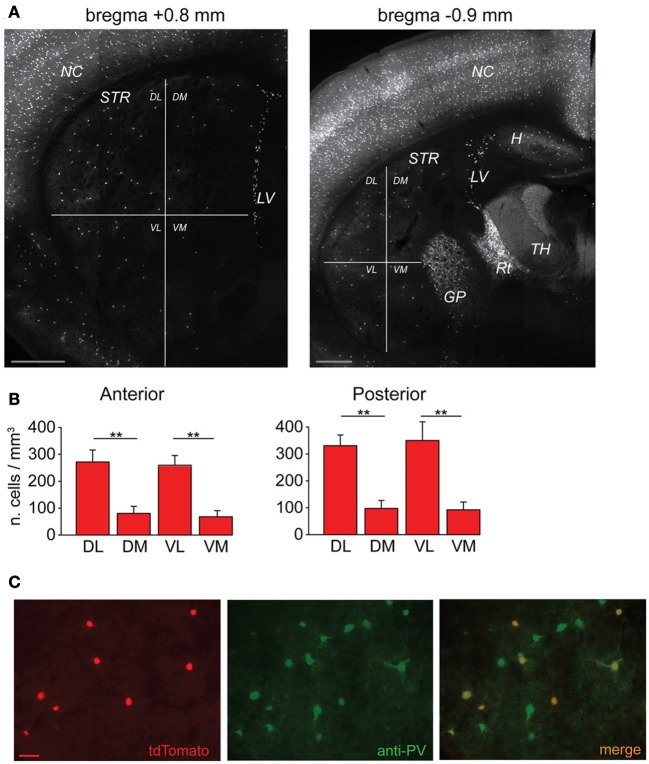
**Anatomical distribution of striatal FSIs and specificity of parvalbumin expression**. **(A)** PV^+^-tdTomato-labeled FSIs in mouse acute slices. FSIs were prevalently distributed in the dorsolateral (DL) and ventrolateral (VL) sectors of the striatum (STR) as compared to dorsomedial (DM) and ventromedial (VM) quadrants. Examples of anterior (left) and posterior (right) 50 μm-thick striatal sections are shown (+0.8 mm and −0.9 mm from bregma, respectively). Note the greater density of tdTomato labeled cells in other areas such as neocortex (NC), globus pallidus (GP), reticular thalamic nucleus (Rt) and lateral ventricles (LV), and hippocampus (H), while non-reticular thalamic nuclei (TH) contain densely stained fibers but are almost entirely devoid of tdTomato-labeled cell bodies. Scale bars: 500 μm. **(B)** Quantification of FSI distribution in the striatum. Data refer to average cell densities per slice ± s.e.m. Counts were made in 50 μm thick slices (*n* = 19) and grouped according to anterior vs. posterior localization of the relative slice with respect to bregma (see Results). Anterior: *n* = 8 slices, ^**^p < 0.01 paired *t*-test; posterior: *n* = 11 slices, ^**^p < 0.01. **(C)** Comparison between expression of td-Tomato (left) and anti-PV immunostaining (middle) in a sample area of DL striatum. An overlap of the two images (right) shows that all td-Tomato cells were also PV-immunopositive, while roughly 50% of the PV-immunopositive cells did not express tdTomato. Scale bar: 50 μm.

Patch-clamp recordings were performed on pairs of genetically identified FSIs in striatal slices prepared from a recombinant Cre-lox mouse line obtained by crossing 129P2-Pvalbtm1(cre)Arbr (JAX stock number: 008069) and 129S6-Gt(ROSA)26Sortm14(CAG-tdTomato)Hze mice (JAX stock number: 007914; Jackson Laboratory, Bar Harbor, ME, USA; Madisen et al., 2010). Offspring mice, which appeared viable and healthy, expressed the td-Tomato red fluorescent protein in FSIs throughout the brain (Figure 1). All procedures were approved by the Italian Department of Health and were conducted in accordance to FELASA guidelines as well as Italian and European directives (DL 116/92 and 2010/63/EU). Mice of both sexes (p18–p38, average p25) were anesthetized with an intraperitoneal injection of a mixture of ketamine/xylazine (100 mg/kg and 10 mg/kg, respectively) and perfused transcardially with ice-cold artificial cerebrospinal fluid (ACSF) consisting of (in mM): 125 NaCl, 2.5 KCl, 1.25 NaH_2_PO_4_, 2 CaCl_2_, 25 NaHCO_3_, 1 MgCl_2_, and 11 D-glucose, saturated with 95% O_2_ and 5% CO_2_ (pH 7.3). After decapitation, brains were removed from the skull and 300 μm-thick parasagittal slices were cut in ACSF at 4°C using a VT1000S vibratome (Leica Microsystems, Wetzlar, Germany). Individual slices were submerged in a recording chamber in which ACSF was continuously flowing (1–2 ml/min) at 32°C. NBQX (5 μ M) was added to ACSF at the beginning of experiments to block AMPA receptors. Patch clamp glass pipettes (2–4 MΩ) contained the following (in mM): 10 NaCl, 124 KH_2_PO_4_, 10 HEPES, 0.5 EGTA, 2 MgCl_2_, 2 Na_2_-ATP, 0.02 Na-GTP, (pH 7.2, adjusted with KOH). For whole-cell recordings in high intracellular chloride conditions and for perforated-patch experiments the following solution was used (in mM): 10 NaCl, 24 KH_2_PO_4_, 100 KCl, 10 HEPES, 0.5 EGTA, 2 MgCl_2_, 2 Na_2_-ATP, 0.02 Na-GTP (pH 7.2, adjusted with KOH). For perforated-patch recordings the intracellular solution was added with gramicidin D (final concentration 5–10 μg/ml, from a stock solution of 5 mg/ml dissolved in DMSO), a ionophore compound which creates Cl^−^-impermeable membrane pores (Ebihara et al., 1995). Recording pipettes were tip-filled with a gramicidin-free solution and back-filled with a gramicidin-containing solution. A stable perforated-patch was achieved after obtaining a gigaohm seal and waiting 30–40 min until the access resistance had gradually reached a stable value (<30 MΩ). Capacitive currents were continuously monitored by applying voltage pulses (−10 mV, 500 ms) from a holding potential of −70 mV. Recordings were discarded when a sudden membrane rupture occurred as indicated by a quick increase in the amplitude of capacitive transients and a rightward shift in inhibitory postsynaptic current (IPSC) reversal potential. Voltage- and current clamp recordings were performed using a MultiClamp 700B amplifier interfaced to a PC through a Digidata 1440A (Molecular Devices, Sunnyvale, CA, USA). Series resistance was compensated in current clamp recordings using the bridge-balance control of the amplifier software panel.

### Data acquisition and analysis

Data were acquired at a sampling frequency of 10 KHz and filtered at 2 KHz using pClamp10 software (Molecular Devices) and analyzed with Origin 8.5 (Origin Lab, Northampton, MA, USA). Immediately after obtaining a whole-cell configuration in voltage-clamp mode in two cells, recordings were switched to current-clamp and the resting membrane potential was measured. The membrane time constant was calculated by fitting with a single exponential equation the rising trajectory of the membrane voltage response to an injected rectangular current pulse (−100 pA, 500 ms). The spike threshold was measured at the time point at which the first derivative of the AP waveform rapidly deflected upwards. The spike width was defined as the time window at half-maximal spike amplitude.

The coupling coefficient (CC) between FSI pairs connected through GJ was calculated after injecting a hyperpolarizing current step (−300 pA, 500 ms) in one cell and dividing the steady-state value of the voltage deflection observed in the non-injected cell by that measured in the injected one. Gc was calculated according to the following equation (Galarreta and Hestrin, 2002):

(1) $$\text{Gc}=1/\left[(R_{\text{in}}/\text{CC})-R_{\text{in}}\right]\text{​},$$

where *R*_in_ is the input resistance of the non-stimulated cell and CC is the coupling coefficient. The input resistance was measured in each cell by dividing the steady-state value of the voltage deflection evoked in response to a hyperpolarizing current step (−100 pA, 500 ms) by the amplitude of the injected current. The amplitudes of APs and afterhyperpolarizations (AHPs) were measured as the voltage difference between spike positive and negative peak vs. spike threshold, respectively.

Unitary spikelet peak and trough amplitudes were measured in current clamp-mode during firing trains at 30–40 Hz, using the point of slope change in uprise deflection as a baseline reference (Figure 3A, inset). The temporal summation of spikelet hyperpolarizing phases (SHPs) was calculated as the ratio between any largest SHP peak and the first SHP peak during a spikelet train at 10–120 Hz. The reference baseline was the average membrane potential value (V_m_) across a 5 ms window preceding the first spikelet. Paired-pulse ratios of unitary GABAergic currents were measured by eliciting two IPSCs at a time interval of 50 ms and calculating the ratio between the second IPSC peak amplitude and the first one. Since fast GJ currents (I_GJ_) were partially overlapped to the rising phase of IPSCs in pairs connected through both electrical and GABAergic synapses (Figure 5A, inset), IPSC 10–90% rise time values were measured only in a subset of pairs which were connected exclusively through GABAergic synapses. Conversely, IPSC peak amplitude and decay time constant values were well separated from I_GJ_, therefore these values were collected from both GJ + GABA and GABA-only pairs. The reversal potential for Cl^−^ ions was calculated using the Nernst equation, *E*_Cl_ = −60log[Cl]_o_/[Cl]_i_ at *T* = 32°C.

The frequency-dependent temporal summation of SHPs (Figure 3A) was fit using the Boltzmann equation:

(2) $$y=A2+\frac{A1-A2}{1+e^{\frac{x-\;x_{0}}{dx}}}$$

where *A1* and *A2* represent the left- and rightmost asymptotic values of *y*, respectively, *x*_0_ is the frequency value corresponding to half-maximal summation, and *dx* is the rising phase slope.

The degree of covariation in firing activities of connected cells was quantified by means of the Pearson's correlation coefficient (PMFR), which was computed according to equation 3:

(3) $$PMFR=\frac{\sum_{i}\left(MFR_{1}(t_{i})-\bar{MFR_{1}})(MFR_{2}(t_{i})-\bar{MFR_{2}}\right)}{\sqrt{{\sum_{i}\left(MFR_{1}(t_{i})-\bar{MFR_{1}}\right)}^{2}{\sum_{i}\left(MFR_{2}(t_{i})-\bar{MFR_{2}}\right)}^{2}}}$$

where *MFR*_1_(*t*_*i*_) and *MFR*_2_(*t*_*i*_) correspond to the time-varying mean firing rate of the two cells measured at the instants *t*_*i*_ and *MFR*_1_, *MFR*_2_ represent the average firing rates. Time-dependent *MFRs* were computed on sliding time windows of fixed size (150 ms) stepped by *t*_*i*_ intervals of 10 ms. The size of the sliding window was large enough to ensure a reliable estimation of the firing rates and the *PMFR*. The co-silent instants (i.e., *MFR*_1_(*t*_*i*_) = *MFR*_2_(*t*_*i*_) = 0) were discarded from the analysis to prevent positive biases of the *PMFR* when both cells were not firing. *PMFR* values varied in the interval [−1,1], where values > 0 indicate positive covariation while values < 0 indicate negative covariation of the firing activities.

Cross-correlations were computed according to equation 4:

(4) $$C_{12}(\tau )=\frac{\sum_{t_{i}}X_{1}(t_{i})X_{2}(t_{i}+\tau )-<X_{1}X_{2}>}{\sqrt{N_{1}N_{2}}}$$

where *t*_*i*_ is the spike time of the reference spike train *X*_1_ (*i* = 1..*N*_1_) with time resolution 1 ms and the convention *X*_1_(*t*_*i*_) = 1 or 0, depending on whether a spike did or did not occur in the time bin *t*_*i*_, respectively. < *X*_1_*X*_2_ > is the joint average number of spikes. The normalization factor is the geometric mean of the number of spikes in the two trains (*N*_1_ and *N*_2_). A threshold for significant cross-correlation was determined as the mean plus two standard deviations of cross-correlation peaks computed by jittering the original spike train (maximal shift equal to the mean inter-spike-interval). PMFR and cross-correlation analyses were performed by means of a custom-made code developed in Python (www.python.org).

Statistical analyses were performed using paired or unpaired Student's *t*-tests for normally distributed data sets, and Wilcoxon signed rank test otherwise (SigmaStat; Systat Software, Chicago, IL, USA). Results are given as means ± s.e.m. in text and represented by box plots in figures. Boxes include 25th and 75th percentiles (horizontal edges), median value (inner line), and min-max values (whiskers). Differences were considered significant at *p* < 0.05.

### Dynamic-clamp

Simulated EPSC conductances (sEPSCs) were injected through the patch pipette using a SM-2 Digital Conductance Injection System (Cambridge Conductances, Cambridge, UK) interfaced through a P25M DSP board (Innovative Integration, Simi Valley, CA, USA). sEPSCs were modeled in Python based on the waveform of real spontaneous EPSCs recorded in FSIs in voltage-clamp mode at a *V*_clamp_ of −70 mV. The 10–90% rise time and decay time constant (τ_dec_) were 0.3 ms and 0.85 ms, respectively. Peak conductance amplitudes (6–8 nS) were set to values which were suitable to bring V_m_ to firing threshold during high-frequency stimulation in current-clamp mode. The reversal potential was set at 0 mV.

### Immunohistochemistry

Mice were anaesthetized with ketamine/xylazine (100 mg/kg and 10 mg/kg, respectively) and perfused transcardially with 4% paraformaldehyde in 0.1 M phosphate buffer at pH 7.4. Brains were quickly removed and placed in fixative overnight at 4°C. Subsequently they were cryo-protected in 30% sucrose in 0.1 M phosphate buffer overnight at 4°C. Brains were rapidly frozen and 50 μm thick coronal sections were cut using a Microm HM450 sliding microtome (Thermo Fisher Scientific, Waltham, MA, USA). Sections were rinsed three times with 1% TBS and stored for 2 h in a blocking solution (1% TBS + 0.5% Triton + 1% BSA + 5% NGS) at 4°C. Eventually, sections were incubated with a rabbit polyclonal anti-parvalbumin (PV) antibody (1:600) overnight at 4°C, rinsed three times in 1% TBS + 0.1% Triton, and subsequently incubated with a goat anti-rabbit Alexa 488-conjugated secondary antibody (1:200; Life Technologies, Monza, Italy) for 3 h in a dark room at 4°C. Sections were then mounted in Vectashield (Vector Laboratories, Peterborough, UK) and examined with a fluorescence BX51 microscope (Olympus, Japan) equipped with a reconstruction software (Neurolucida, MBF Bioscience, Magdeburg, Germany). For each slice the striatum was subdivided in four sectors (dorso- and ventrolateral, dorso- and ventromedial) and the volume of each sector was measured using the Neurolucida image reconstruction system. Fluorescent FSIs were counted in every sector across three sequential focal planes (each of 15 μm along the Z axis) acquired using confocal laser scanning. Cell densities were obtained by dividing the number of FSIs by the volume of the relative sector.

For cell reconstruction the intracellular solution was added with Neurobiotin 488 tracer (1.5 mg/ml; Vector Laboratories, Peterborough, UK). Individual FSIs were patch-clamped in whole-cell configuration and their firing pattern was recorded in current-clamp mode. After recording, slices (300 μm of thickness) were fixed overnight at 4°C in 4% paraformaldehyde in 0.1% phosphate buffer (pH 7.4). Eventually, sections were rinsed three times with 4% PBS, mounted in Vectashield, and examined with a confocal SP5 upright microscope (Leica, Germany).

### Drugs

All drugs were obtained from Sigma except NBQX, SR95531, and CGP52432 (Abcam Biochemicals, Bristol, UK).

## Results

**Figure 2:**
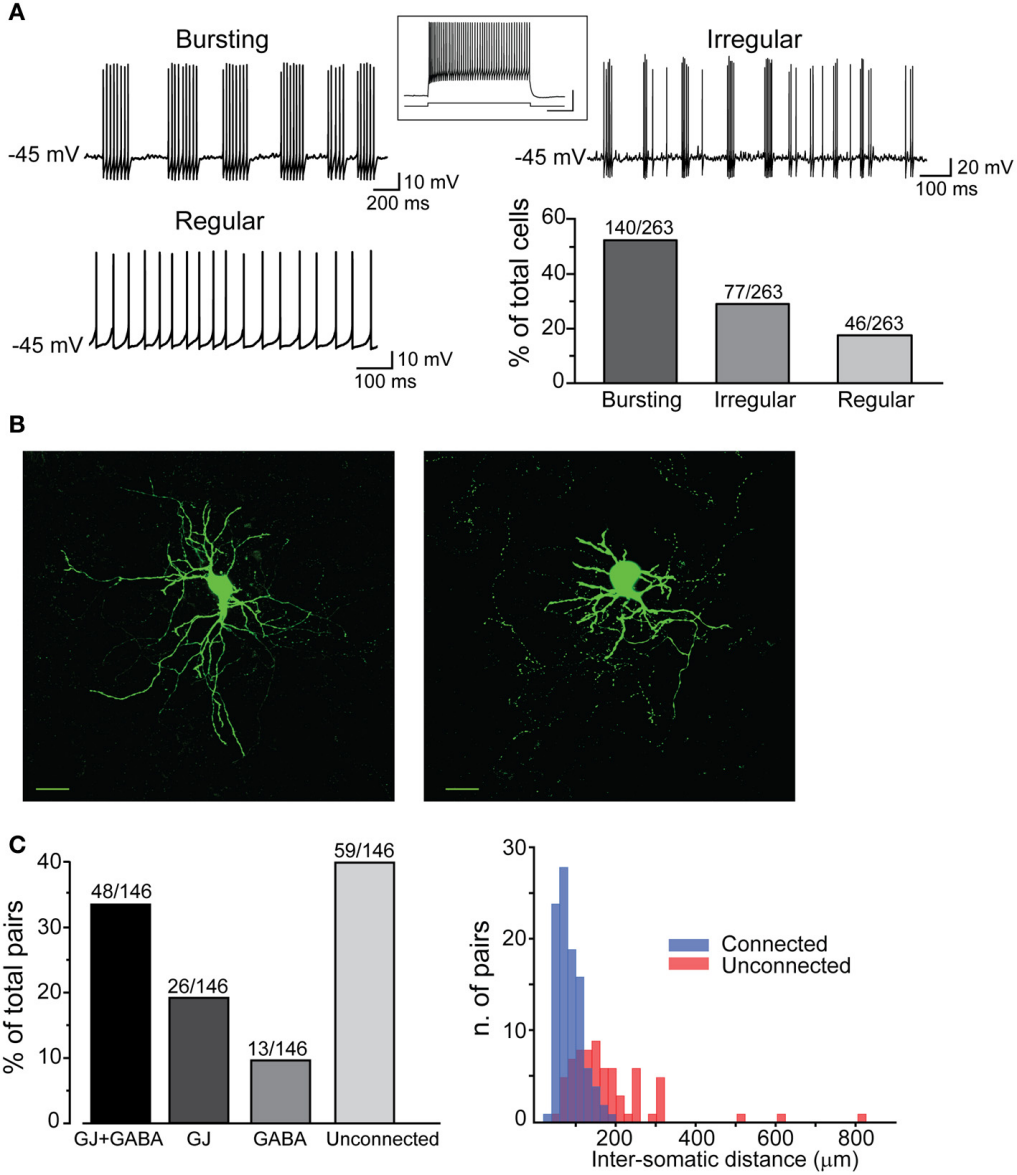
**Electrophysiological properties and connectivity patterns of striatal FSIs. (A)** Examples of different firing patterns and relative distributions. Traces are excerpts from 20 to 60 s lasting recordings in which cells were injected with suprathreshold DC (I_inj_ bursting: 500 pA; regular: 400 pA; irregular: 500 pA). The histogram shows relative distributions of different firing patterns. Inset: high-frequency firing activity in response to supra-threshold current pulse (700 pA, bottom trace). Calibration: 30 mV, 250 ms. **(B)** Confocal microphotographs showing two different FSIs filled with neurobiotin-488 (scale bars: 20 μm). **(C)** Left, rates of different connectivity patterns between pairs of striatal FSIs. Right, spatial distribution of connected and unconnected FSI pairs relative to the distance between cell bodies.

In order to visualize PV-expressing interneurons we used striatal slices obtained from transgenic mice in which the fluorescent protein tdTomato was expressed under the promoter for PV (see Methods). FSIs were visible in the striatum as a relatively sparse population of tdTomato-labeled cells (Figure 1A) significantly more abundant in lateral than medial striatal areas, both anteriorly and posteriorly (up to +1.2 mm and −1.2 mm from bregma, respectively; Figure 1B) consistently with previous reports (Gerfen et al., 1985; Kita et al., 1990; Mura et al., 2000; Luk and Sadikot, 2001). After immunohistochemical staining with an anti-PV antibody, the fraction of labeled cells which also expressed tdTomato was 40 ± 3% of the total anti-PV labeled cells (238 anti-PV^+^/tdTomato^+^ vs. 602 anti-PV^+^/ tdTomato^−^, *n* = 19 slices from 4 mice), while 100% of tdTomato-expressing cells were labeled by anti-PV antibodies (Figure 1C). The high specificity of the reporter gene expression was confirmed by the electrophysiological properties of recorded cells, which invariably displayed typical patterns of FSIs (see below). For recordings, we used cell pairs located mainly in the anterior or posterior dorsolateral portion of the striatum. No significant differences were found between electrical properties or connectivity patterns of FSIs located in different striatal territories, therefore data were pooled together. Simultaneous whole-cell patch clamp recordings were performed in pairs of tdTomato-labeled FSIs. Intrinsic membrane and spike properties of these cells are summarized in Table 1. In current-clamp experiments most FSIs responded to injection of relatively brief, strong current pulses (700 pA, 500 ms) with a regular train of AP at a mean frequency of 132 ± 2 Hz (Figure 2A, inset). To detect steady-state firing patterns in a subset of FSIs we injected prolonged suprathreshold DC (I_inj_: 400–800 pA, 20–150 s; Figure 2A). Most cells (140 out of 263, 53%) responded with repetitive bursts of AP with a mean intra-burst frequency of 39 ± 1 Hz. A minor subset of FSIs (46 out of 263, 18%) fired in single-spike mode (mean frequency 17 ± 1 Hz), while another group (77 out 263, 29%) fired with an irregular alternation of short bursts and single spikes (mean frequency 6 ± 0.5 Hz). In fifteen neurobiotin-filled FSIs reconstructed with a confocal microscope the different firing patterns did not specifically correspond to diverse morphologies, i.e., bursting, single-spike, or irregularly firing cells displayed dendritic fields which were either relatively spread (180–280 μm of diameter centered at the soma; *n* = 8) or more confined (100–120 μm; *n* = 7; Figure 2B).

**Table 1:** **Electrophysiological properties of striatal FSIs**.

	**Bursting (*n* = 140)**	**Irregular (*n* = 77)**	**Regular (*n* = 46)**
Resting membrane potential (mV)	−74 ± 0.5	−74 ± 0.6	−76 ± 1
Input resistance (MΩ)	60 ± 2	63 ± 3	59 ± 5
Membrane time constant (ms)	9.8 ± 0.2	9.5 ± 0.4	9.3 ± 0.4
AP peak amplitude (mV)	60 ± 1	55 ± 2	58 ± 2
AP threshold (mV)	−45 ± 0.6	−43 ± 0.5	−46 ± 1
AP width (ms)	0.54 ± 0.01	0.56 ± 0.01	0.55 ± 0.02
AP time to peak (ms)	0.44 ± 0.01	0.46 ± 0.01	0.46 ± 0.02
AHP amplitude (mV)	15 ± 0.4	14 ± 0.5	13 ± 0.7

All values are means ± s.e.m. Input resistance and membrane time constant were measured on passive voltage responses to injection of negative current pulses (−100 pA, 500 ms). Action potentials (APs) were elicited by injection of suprathreshold current pulses (400 pA, 500 ms). AHP: afterhyperpolarization. AP time to peak and AHP were calculated using the spike threshold as a reference point.

**Figure 3:**
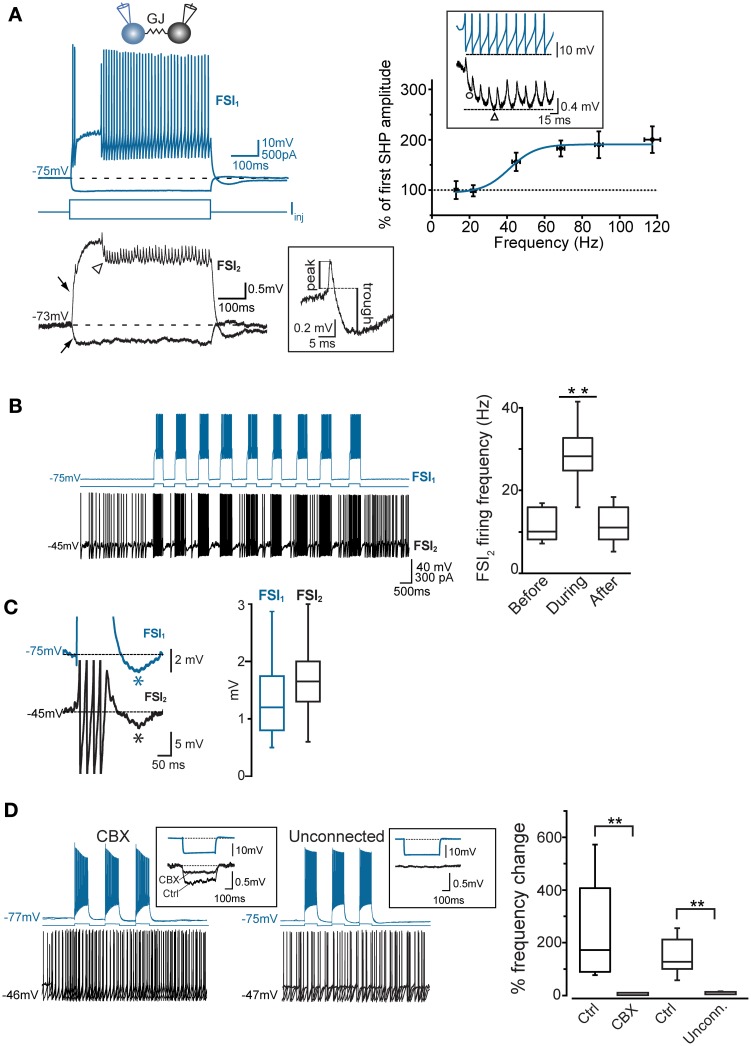
**Coordination of firing activity in electrically connected FSIs**. **(A)** Top, voltage responses of FSI_1_ during injection of hyper- and depolarizing current steps (−300 and +400 pA, respectively, 500 ms). Bottom, passive responses mediated by GJ in FSI_2_ (arrows). Spikelets were elicited in FSI_2_ during AP firing in FSI_1_. Note temporal summation of spikelet hyperpolarizing potential (SHP, arrowhead). The inset shows an individual spikelet with reference points used for calculating peak and trough amplitudes. Top right panel, frequency-dependence of SHP temporal summation. Each data point represents the average ratio between the amplitude of the most hyperpolarized SHP (marked by a triangle in the inset lower trace) and the amplitude of the first SHP in the array (circle). To reduce data scattering, ratios were grouped within 15–35 Hz spanning segments along the x-axis (each segment contained 10–15 data points from a total of 38 pairs). A Boltzmann fit of the data set (blue line; see Methods) yielded the following parameters: A1 = 0.95 ± 0.2, A2 = 1.9 ± 0.1, *x*_0_ = 41.2 ± 5.4 mV, and *dx* = 7.0 ± 6.3. Note how spikelet maximal summation was delayed with respect to maximal summation of presynaptic spike AHPs (upper trace in the inset). **(B)** Left, example of transient, repetitive firing entrainment of two electrically connected FSIs (see text for details). Right, summary of average postsynaptic firing frequencies before, during, and after the injection of current pulses in FSI_1_ (*n* = 48 pairs, ^**^p < 0.01, paired-sample *t*-test). **(C)** Left, magnified view of individual burst events occurring in two FSIs in response to a suprathreshold current pulse injected in FSI_1_. Action potential firing was graphically removed in FSI_1_ and partially truncated in FSI_2_ in order to emphasize after-hyperpolarization (AHP, marked by asterisks). Right, summary box plots of AHP amplitudes in FSI_1_ and FSI_2_. **(D)** Lack of firing co-activation in response to injection of suprathreshold current pulses in FSI_1_ (blue) either in the presence of 200 μ M carbenoxolone (CBX) or in unconnected pairs. Insets show V_m_ changes in FSI_2_ in response to a hyperpolarizing current pulse (−300 pA, 500 ms) injected in FSI_1_. The box chart on the right shows a statistical summary relative to frequency changes during current injection in FSI_2_ in control conditions, after bath application of CBX (*n* = 8, ^**^p < 0.01, paired *t*-test) and in connected vs. unconnected pairs (^**^p < 0.01, unpaired *t*-test, *n* = 12).

All different types were used to analyze membrane properties, connectivity rates, and functional interactions mediated by GJ and GABAergic synapses. Electrical coupling combined with GABAergic synapses was found in 48 out of 146 pairs of FSIs (33%) recorded under control conditions (i.e., ACSF + 5 μ M NBQX; Figure 2C, left panel). Electrical synapses alone were detected in 18% of total pairs (23% bidirectionally and 77% unidirectionally). Another 9% of total pairs (13 out of 146) were connected via GABAergic synapses only (31% bidirectionally and 69% unidirectionally), while the remaining 40% were unconnected. The somata of pairs that were connected through any of the three patterns described above (i.e., GJ + GABA; GJ alone; GABA alone) were relatively close to each other (82 ± 2 μm), whereas pairs with somata separated by larger distances (up to 800 μm; average 179 ± 15 μm) were mostly unconnected (Figure 2C, right panel). Thus, FSIs were highly connected to their proximal neighbors but not to more distant cells. Properties of GJ-mediated electrical coupling are shown in Figure 3A. Upon injection of hyperpolarizing current steps in one of two simultaneously recorded cells both interneurons responded with voltage deflections corresponding to a CC of 0.06 ± 0.003 (*n* = 74 pairs), yielding a mean Gc of 1.3 ± 0.1 nS (see Methods). CC and Gc values were usually asymmetrical within each pair (i.e., CC_FSI1 → FSI2_ ≠ CC_FSI2 → FSI1_). The average smaller value across all pairs was significantly different from the average larger value (CC_small_0.045 ± 0.005, CC_large_0.075 ± 0.008, *n* = 68, *p* < 0.05, paired *t*-test; Gc_small_1.2 ± 0.1 nS, Gc_large_1.7 ± 0.2 nS, *n* = 68, *p* < 0.05, paired *t*-test). When suprathreshold depolarizing current pulses were injected in FSI_1_ to induce firing of AP trains, FSI_2_ responded with arrays of fast spikelets which were time-locked to AP in FSI_1_. Spikelet properties were measured during presynaptic AP trains at a frequency of 30–40 Hz in the presence of the GABA_A_ receptor antagonist gabazine (10 μ M). Spikelets were composed by a small, relatively fast depolarizing phase having a mean peak amplitude and time-to-peak of 0.41 ± 0.04 mV and 1.44 ± 0.1 ms, respectively, followed by a slower hyperpolarizing trough (mean amplitude and time-to-trough: 0.53 ± 0.06 mV and 10.9 ± 0.6 ms, respectively). The spikelet positive peak followed the presynaptic spike peak by a delay of 0.9 ± 0.1 ms. Spikelets were reliably evoked by every individual AP across lengthy periods of firing. Temporal summation of presynaptic AP AHPs propagated in a low-pass filtered form to the coupled FSI so that a net V_m_ hyperpolarization occurred within the first 100 ms (mean 73 ± 3 ms, *n* = 38 pairs) from the negative peak of the first spikelet evoked at the onset of a presynaptic AP train (Figure 3A). Temporal summation was absent at frequencies <25 Hz, but steeply rose to a maximum in a range between 60 and 120 Hz.

To investigate the interplay between firing activities of FSI pairs connected via electrical and/or chemical synapses, we depolarized V_m_ in current-clamp mode to near-threshold values by injecting steady DC (I_inj_: 300–500 pA) in one of the two cells (FSI_2_ in Figure 3B) in order to elicit AP firing at a relatively low frequency (6.2 ± 1.3 Hz, *n* = 48 pairs). When FSI_1_ was also injected with short, repetitive suprathreshold current pulses (200–500 ms, 600 pA), charge coupling through GJ induced a small additional depolarization in FSI_2_ which in turn increased significantly its firing frequency in correspondence of each current step (30.1 ± 2 Hz, *n* = 48 pairs, *p* < 0.001, paired-sample *t*-test; Figure 3B, right). When FSI_1_ stimulation was ceased, the mean firing frequency in FSI_2_ returned to an average value similar to that preceding the stimuli (7.4 ± 2.0 Hz, *n* = 48 pairs, *p* > 0.05, paired-sample *t*-test). Notably, as each of the current pulses was set off the post-firing AHP induced in FSI_1_ (average 1.3 ± 0.2 mV) was matched by an AHP in FSI_2_ (average 1.9 ± 0.3 mV; Figure 3C) which was often sufficient to cause a transient pause in firing. Thus, the simultaneous occurrence of a depolarizing drive followed by an AHP in both cells contributed to entrain them into a pattern of firing co-activity. To test the involvement of electrical synapses in pair co-activation we perfused slices with the GJ blocker carbenoxolone (CBX, 200 μ M; Figure 3D) for 30–60 min. In order to prevent unspecific effects by CBX (Tovar et al., 2009), recordings were performed in the presence of AMPA- and GABA_A_ receptors antagonists NBQX (5 μ M) and gabazine (10 μ M), respectively. CBX also affects NMDA receptors (Chepkova et al., 2008; Tovar et al., 2009); however, these are not expressed in striatal FSIs (Gittis et al., 2010). In our experiments CBX did not significantly change individual spike waveforms (see below) and was preferred to mefloquine (a more specific blocker of Cx36 GJ protein; Cruikshank et al., 2005) because of a relatively faster inhibitory action by CBX and because long incubations with 25–100 μ M mefloquine (>2 h), besides inducing an expected reduction of the CC, caused a block of repetitive AP firing upon prolonged DC injection, precluding the detection of GJ-mediated effects on spike activity (not shown). CBX caused a 64 ± 4% reduction in CC with respect to control conditions (Figure 3D, left inset; *n* = 8, *p* < 0.05, paired *t*-test). We also recorded unconnected pairs in which no baseline deflection occurred in response to presynaptic current injection (Figure 3D, right inset). As expected, firing co-activation was completely absent either in non-connected pairs or in GJ-connected pairs after CBX perfusion (Figure 3D, right panel).

**Figure 4:**
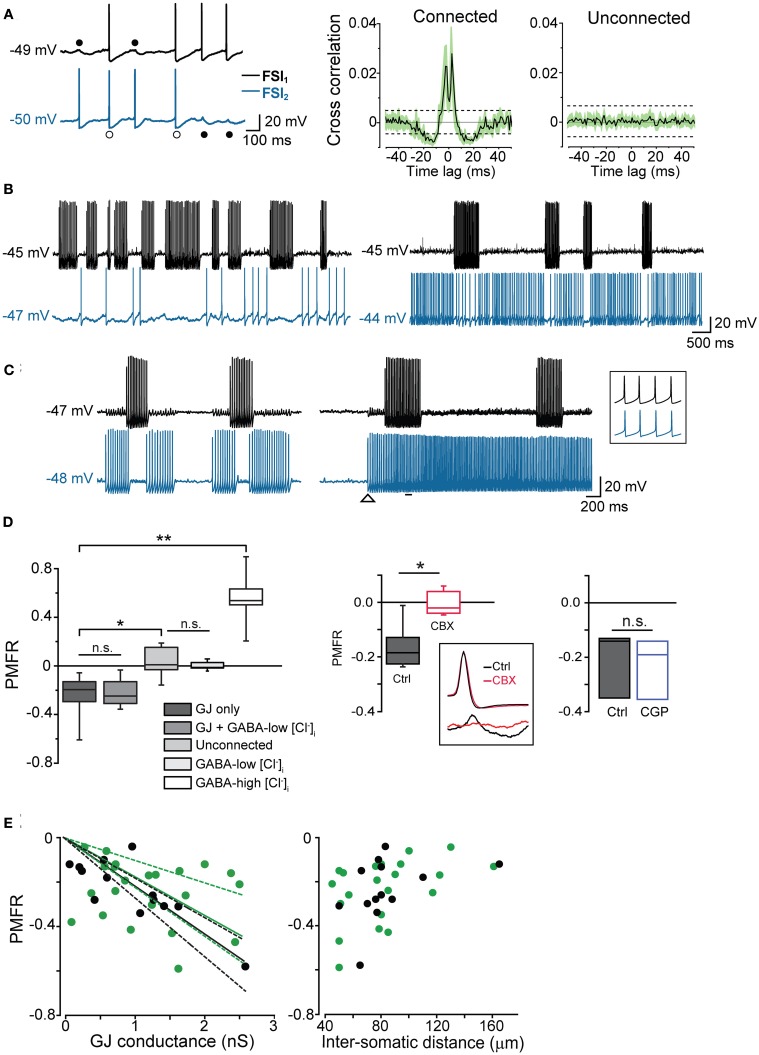
**GJ mediate inhibition in coupled interneurons at high-frequency presynaptic spike firing**. **(A)** In order to induce prolonged episodes of firing activity at low frequency (in this case 8 Hz in both cells), two electrically connected FSIs (without GABAergic synapses) were injected with a relatively small suprathreshold DC (200–250 pA) through the recording electrodes for 1–2 min. Two-second recording segments are shown here starting 5–10 s after the onset of current injection. Left, individual spikes either elicited an AP (white circles) or a spikelet (black circles) in the paired FSI. Right, average cross-correlograms for connected and unconnected pairs (*n* = 11 and 6, respectively). Shaded areas and dashed lines represent s.e.m. and average confidence intervals equal to two standard deviations of the spike trains, respectively **(B)** Left, burst-like firing episodes in FSI_1_ (I_inj_: 430 pA; intra-burst frequency: 38 Hz) were associated with silent periods in FSI_2_ when the latter was stimulated just above firing threshold (I_inj_: 310 pA; average spike frequency: 1.8 Hz). Right, in the same pair, FSI_2_ was injected with stronger DC (I_inj_: 370 pA) to increase the firing frequency (~20 Hz). Spike trains in FSI_1_ were still able to induce a reduction of firing activity in FSI_2_. **(C)** Left, spike inhibition resulted in alternated burst firing in two electrically connected FSIs. Right, in the same pair, the inhibitory effect was overrun by a strong increase in firing frequency in FSI_2_ (from 29 to 52 Hz; I_inj_ was increased from 380 to 430 pA at the instant indicated by the arrowhead). The inset shows synchronous spikes in a magnified time window indicated by the horizontal bar. **(D)** Left, box-plot summary of Pearson's mean firing rate coefficient values (PMFR) for connected and unconnected FSI pairs (^*^p < 0.05, ^**^p < 0.01, n.s.: not significant at *p* > 0.05, unpaired *t*-test). Right, box plot summarizing the effect of CBX (200 μ M) and CGP52432 (5 μ M) relative to paired controls (^*^p < 0.05, *n* = 5; n.s.: *p* > 0.05, *n* = 3, Wilcoxon signed rank test). The inset shows the inhibitory effect of CBX on spikelet (bottom) but not AP waveforms (top). **(E)** PMFR values plotted against GJ conductance (left) and inter-somatic distance (right) for pairs connected by GJ-only (green circles) and GJ + GABA (black circles). Data sets are the same as the ones used for box plots in **(D)**. Solid lines are linear regression fits of the respective data sets (dashed lines represent confidence bands at 95% level).

These data indicate that GJ-coupled FSIs were entrained into episodes of concurrent firing activity paced by the rhythm of the stimulating current pulses injected in one cell. In order to investigate in more detail the relationship between firing trains, we extended the period of supra-threshold current injection (200–600 pA) in both cells up to 1–2 min in 47 pairs (Figure 4). During spike trains characterized by relatively low firing rates (2–20 Hz, with each of the two cells firing at the same or nearly equal average frequency), some spikes were tightly synchronized whereas others induced a postsynaptic spikelet followed by a hyperpolarizing phase (SHP; Figure 4A). Cross-correlation analysis revealed two positive peaks around zero lag (−2.4 ± 0.5 ms and 2.2 ± 0.2 ms, respectively, *n* = 11) and negative troughs at larger lags (−14.3 ± 1.3 ms and 13.7 ± 2.1, respectively, *n* = 11; Figure 4A, middle panel), indicating that both spike synchronization and inhibition occurred during low-frequency firing activity. The average cross-correlogram in unconnected pairs did not show significant peaks (Figure 4A, right). At higher frequencies, however, AP firing appeared largely inhibited, i.e., spike bursts occurring in one cell were concomitant with silent periods in the other one (Figure 4B). This phenomenon was particularly evident in pairs where one FSI was bursting while the other displayed a regular or irregular single-spike firing mode (Figure 4B). In these pairs, high-frequency bursts (25–60 Hz) occurring in FSI_1_ were often highly effective in silencing or slowing the firing activity of FSI_2_, even when the firing frequency was augmented in the latter by increasing I_inj_ amplitude (Figure 4B, right). Presynaptic bursts were often able to block or interrupt postsynaptic bursts as well (Figure 4C, left), provided that the two cells fired at an intra-burst frequency of less than 50–60 Hz. When both cells exceeded a frequency of 50–60 Hz, however, inhibition failed and the firing became mostly synchronous (Figure 4C, right). In any case, a condition for the occurrence of the latter effect was that at least one cell was tonically firing at high frequency, since we did not observe a plain coincidence of burst activity in two bursting FSIs even when the intra-burst frequency exceeded 50–60 Hz in both cells.

**Figure 5:**
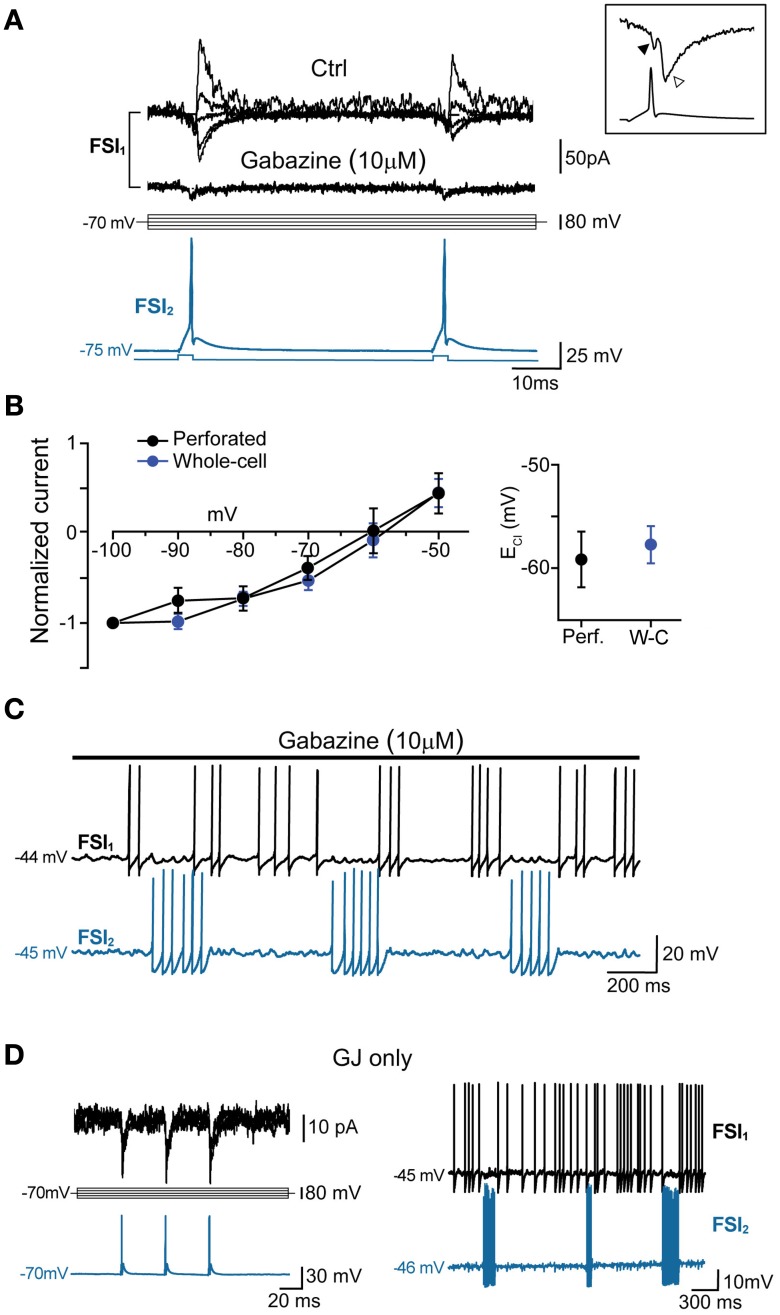
**GABA_A_-mediated fast synaptic transmission is not required for spike inhibition**. **(A)** GABAergic IPSCs (top) evoked in FSI_1_ at various holding potentials (from −100 to −20 mV, 10-mV steps; middle) in response to two consecutive AP elicited in FSI2 at an interval of 50 ms (bottom). In pairs connected by GABAergic synapses, mean peak amplitude, 10–90% rise time, and decay time constant of unitary IPSCs (recorded in voltage-clamp mode at *V*_clamp_ = −90 mV) were −70 ± 14 pA, 0.6 ± 0.08 ms, and 3.7 ± 0.5 ms, respectively, while the mean paired-pulse ratio was 0.85 ± 0.05. Bath application of 10 μM gabazine for 5–10 min completely blocked the IPSCs, unmasking small GJ-mediated currents. Inset, magnification of I_GJ_ (black arrowhead) followed by I_GABA_ (white arrowhead) recorded at −100 mV in response to an individual presynaptic AP (bottom trace) **(B)** Left, current-voltage plots of IPSC amplitudes (mean ± s.e.m.) normalized to average values recorded at −100 mV. Blue and black traces represent data obtained with whole-cell (*n* = 15) and perforated patch recordings (*n* = 4), respectively. Right, summary plot of average reversal potentials for Cl^−^ ions (*E*_Cl_) obtained with perforated patch (−59.6 ± 3 mV, *n* = 4) and whole-cell recordings (−58.2 ± 2 mV; *n* = 15). The theoretical *E*_Cl_ calculated using the Nernst equation in our experimental conditions was −59 mV (see Methods). **(C)** Example of spike series in which inhibition is still evident during application of 10 μ M gabazine. **(D)** Left, voltage-clamp recordings of spikelets alone (i.e., without superimposed IPSCs) at various command potentials in response to three presynaptic AP (blue trace) in a pair connected by GJ, but not GABAergic synapses. Right, mutual inhibition of firing activity recorded in the same pair.

In order to quantify the inhibitory effect mediated by GJ on AP firing, we computed the Pearson Mean Firing Rate coefficient (PMFR; see Methods) for pairs of spike series (20–60 s). A summary of PMFR distributions under different conditions is shown in Figure 4D. The mean PMFR value calculated in GJ + GABA connected pairs was −0.24 ± 0.04 (*n* = 13), corresponding to an inhibitory effect on postsynaptic firing. FSI pairs were often connected through GABAergic synapses (see Figures 5A,B), therefore one might attribute the fast AP inhibition to a GABA-mediated inhibitory effect. However, in GJ + GABA connected pairs we found that spike inhibition remained intact after blocking GABAergic currents through bath application of the GABA_A_ receptor antagonist gabazine (10 μ M; see Figure 5C), or in pairs where GABAergic synapses were undetectable (Figure 5D). The mean PMFR value for FSIs connected exclusively through GJ was −0.23 ± 0.03 (*n* = 22), not significantly different from pairs coupled through GJ + GABA (*p* > 0.3, unpaired *t*-test). In unconnected pairs the mean PMFR value was 0.01 ± 0.05 (corresponding to uncorrelated firing activity), significantly different from both GJ-only and GJ + GABA values (*p* < 0.05, *n* = 12, unpaired *t*-test) but not significantly different from the mean PMFR calculated in five pairs connected exclusively through GABAergic synapses (−0.01 ± 0.01, *p* > 0.05, unpaired *t*-test; Figure 4D) confirming that GABAergic IPSPs did not exert substantial inhibition on postsynaptic firing activity in these cells.

To further validate the ability of PMFR analysis to identify a causal relationship between spike series, we tested whether the algorithm could detect positive covariations in synchronous spike trains. To do so, we performed paired recordings using an intracellular solution containing a high Cl^−^ concentration (114 mM), which resulted in a strong rightward shift in the reversal potential for Cl^−^ ions and thus caused GABAergic postsynaptic potentials (PSP) to be depolarizing at near-threshold voltage levels (Supplementary Figure S1A). Under these conditions, GABAergic PSPs triggered AP in the postsynaptic FSI resulting in a tight synchronization of firing (Supplementary Figures S1B–D). The mean PMFR value obtained in pairs recorded in high [Cl^−^]_i_ conditions was +0.51 ± 0.1 (*n* = 6). This value, representing positively covarying firing rates, was significantly different from all other conditions (*p* < 0.01, unpaired *t*-test).

In five other GJ-connected pairs the mean PMFR values before and after bath application of 200 μ M CBX were −0.15 ± 0.03 and 0.01 ± 0.03, respectively (*n* = 5, *p* < 0.05, Wilcoxon signed rank test; Figure 4D). CBX did not significantly alter the AP shape observed during prolonged DC injection (peak amplitude, ctrl: 54 ± 2 mV, CBX: 53 ± 5 mV; spike width, ctrl: 0.6 ± 0.03 ms, 0.7 ± 0.05 ms; AHP amplitude, ctrl: 18 ± 1 mV, CBX: 18 ± 1 mV; *n* = 10 cells, *p* > 0.05, Wilcoxon signed rank test). Conversely, no postsynaptic spikelets were detected in response to presynaptic AP after CBX application (Figure 4D, inset). Furthermore, the GABA_B_ antagonist CGP52432 (5 μ M) did not affect GJ-mediated inhibition (PMFR ctrl: −0.21 ± 0.07, CGP: −0.23 ± 0.06, *n* = 3, *p* > 0.05, Wilcoxon signed rank test; Figure 4D).

PMFR values from pairs connected through GJ-only or GJ + GABA were linearly correlated with Gc values (Figure 4E, left; the largest conductance value was used for each pair). Despite some divergence, no significant difference was found among linear fits of the two data sets (*F* = 1.45, *p* > 0.2, Origin fit comparison *F-test)*, confirming that GABAergic synapses did not substantially contribute to AP inhibition. Furthermore, PMFR values diminished as the inter-somatic distance of recorded cells increased (Figure 4E, right). Although the scarcity of connected pairs at inter-somatic distances larger than 130–150 μm prevented an appropriate fit of the data sets, these data suggest that spike decoupling had a similar distance-dependence for both GJ-only and GJ + GABA connected pairs.

**Figure 6:**
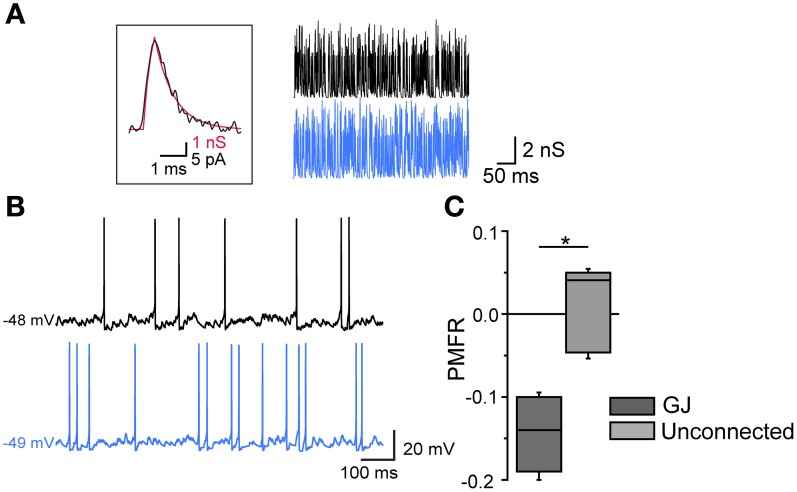
**Gap-junction-mediated firing inhibition in FSI pairs stimulated by arrays of sEPSCs in dynamic-clamp configuration**. **(A)** Left, unitary sEPSC conductance (red trace) superimposed to a unitary spontaneous EPSC (black trace) recorded in FSIs in voltage-clamp mode (*V*_clamp_ = −70 mV; average of 30 EPSCs recorded in 6 different FSIs). The EPSC waveform was reversed and scaled to that of the sEPSC in order to show matching kinetics (10–90% rise time: 0.3 ms; τ_dec_: 0.85 ms). Right, Poisson trains of sEPSCs (300 Hz, 500 ms; segments excerpted from traces lasting 15–20 s) used as dynamic-clamp waveforms. **(B)** Action potential firing in two GJ-connected FSIs stimulated by sEPSC trains shown in **(A)** in the presence of 10 μ M gabazine. **(C)** Summary of PMFR values for GJ-connected and unconnected FSI pairs (*n* = 6, ^*^p < 0.05, Mann-Whitney U test).

Finally, the inhibitory effect by GJ was tested in pairs in which AP firing was induced by injecting poissonian trains of sEPSCs at high frequency (200–500 Hz) in both cells using dynamic-clamp (Figure 6). For each pair the two cells were injected with two uncorrelated sEPSC trains. Trains at higher frequencies (400–500 Hz) were sufficient to supra-linearly depolarize V_m_, while lower frequencies required a relatively small amount of DC injection (50–100 pA) to reach threshold. In both cases, the firing activity was negatively correlated (mean PMFR value in GJ-connected FSIs: −0.15 ± 0.03; unconnected FSIs: 0.02 ± 0.04, *n* = 6, *p* < 0.05, Mann-Whitney U test).

**Figure 7:**
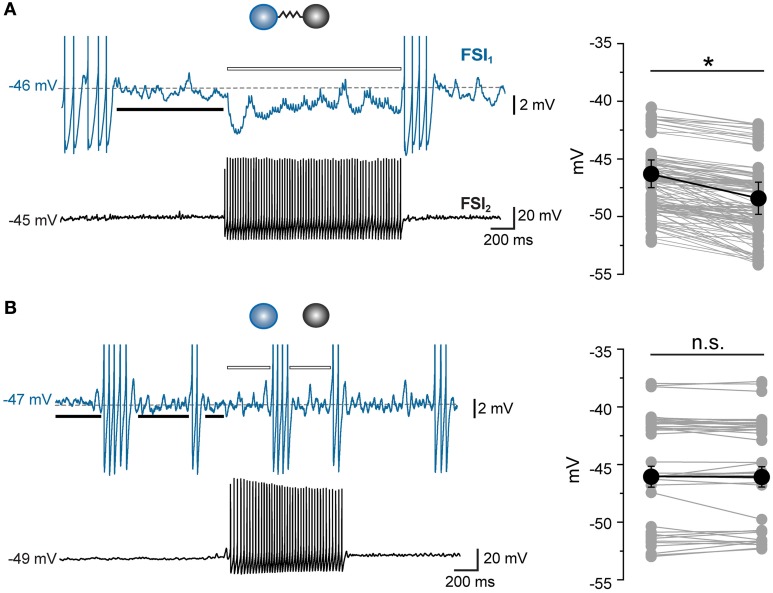
**Gap junctions prevent postsynaptic firing by inducing V_m_ hyperpolarization at near-threshold levels**. **(A)** Example of a GJ-only connected pair in which FSI_1_ displayed an array of subthreshold spikelets in response to a train of AP occurring in FSI_2_ during DC injection in both cells (380 and 405 pA, respectively, 20 s). The average V_*m*_ value of FSI_1_ during the spikelet barrage was measured throughout the duration of FSI_2_ burst (indicated by the white bar) and compared to the average V_m_ value before the burst onset (black bar). Right, summary of average V_m_ values measured in FSI_1_ before (left column) and during (right column) barrages of 4–8 spikelets occurring in response to AP burst in FSI_2_. Mean V_m_ ± s.e.m. values were significantly different in the two conditions (*n* = 22 pairs, ^*^p < 0.05, paired *t*-test). **(B)** Same experiment as in **(A)**, but with non-connected FSIs. Mean V_m_ ± s.e.m. in FSI_1_ did not change significantly during a burst in FSI_2_ (*n* = 10 pairs, *p* > 0.05, paired *t*-test). Transient changes in V_m_ corresponding to AP firing were excluded from measurements.

**Figure 8:**
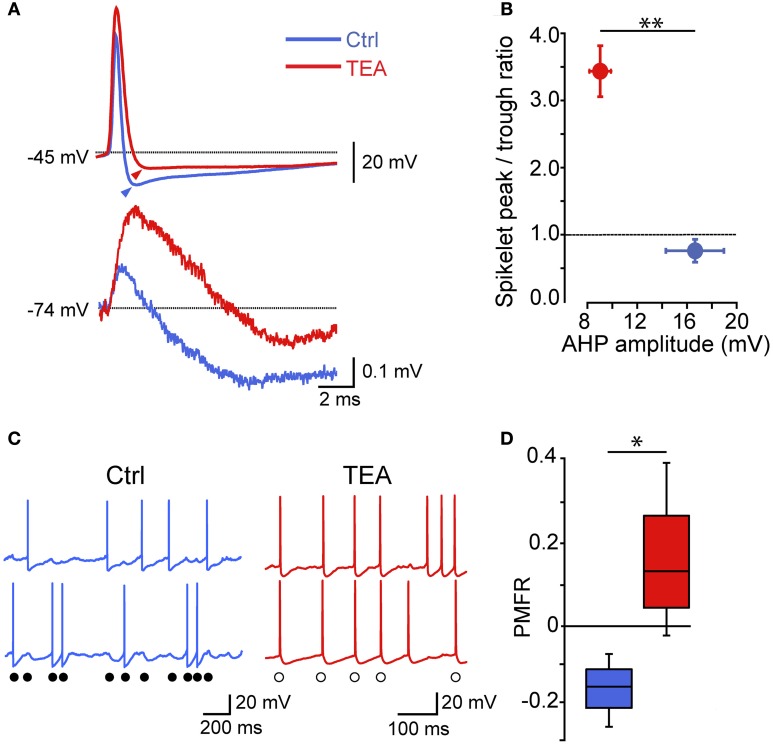
**The spikelet hyperpolarizing phase is determinant for GJ-mediated spike inhibition: effect of reducing a Kv3 conductance**. **(A)** Individual presynaptic AP (top) and postsynaptic spikelets (bottom) recorded in GJ-connected pairs in control conditions and after bath application of 1 mM TEA. Arrowheads indicate AHP peak levels with respect to spike threshold (marked by dotted line). **(B)** Summary of spikelet peak/trough ratios plotted against AHP amplitudes (*n* = 6, ^**^p < 0.01, paired *t*-test). **(C)** Examples of AP trains induced by DC injection (300–400 pA) in ctrl (blue) and after bath application of 1 mM TEA (red). Filled circles indicate spike inhibition in control conditions while empty circles mark synchronized spikes after TEA application. **(D)** Summary of PMFR coefficients switching from negative (ctrl) to positive (TEA) values according to the change in ratio between spikelet peak and trough amplitudes (*n* = 6, ^*^p < 0.05, paired *t*-test).

Thus, electrical synapses in FSI pairs induced two apparently opposite effects: (1) a relatively slow membrane potential coupling, which resulted in a modulation of the firing frequency of one FSI in relatively loose coherence with the other, and (2) a fast AP inhibition causing a mutual firing suppression which could persist during prolonged periods of activity at relatively high frequencies. The ability of GJ to dampen AP firing becomes evident by analyzing V_m_ changes at subthreshold levels during concurrent firing in electrically connected FSIs. As mentioned earlier, trains of spikelets displayed temporal summation of hyperpolarizing phases (Figure 3A; cf. Galarreta and Hestrin, 2001), suggesting that a net inhibitory drive might account for spike silencing during a presynaptic burst. Indeed, trains of spikelets evoked at near-threshold potentials induced an average V_m_ hyperpolarization with respect to a preceding time window in which both cells were silent (−48 ± 1 mV vs. −46 ± 1 mV, respectively, *n* = 22, *p* < 0.05, paired *t*-test, pairs; Figure 7A). Similarly, the mean presynaptic V_m_ level during a train of APs was significantly more hyperpolarized than the V_m_ level preceeding the first AP (−51 ± 1 mV vs. −47 ± 1 mV, respectively, *n* = 22, *p* < 0.05, paired *t*-test). Conversely, in unconnected pairs the average postsynaptic subthreshold potential did not change significantly during presynaptic bursts (−47 ± 1 mV vs. −47 ± 1 mV, respectively, *n* = 10 pairs, *p* > 0.05, paired *t*-test, Figure 7B). Presynaptic mean V_m_ levels before and after AP trains were −45 ± 1 mV vs. −50 ± 1 mV, respectively (*n* = 10, *p* < 0.05, paired *t*-test). Thus, GJ-mediated propagation of presynaptic AHPs into the postsynaptic cell induced a small yet consistent hyperpolarization which was sufficient to decrease the firing probability by turning V_m_ away from the firing threshold (averaging −42 ± 1 mV, *n* = 30 cells). To further investigate the hyperpolarizing effect of GJ we used pharmacological manipulations aimed at increasing the spikelet peak and reducing its trough amplitude in order to reverse the ratio between the two phases (Figure 8). Bath application of TEA at low concentration (1 mM) in the presence of 10 μ M gabazine blocked Kv3-dependent AP repolarizing phase in GJ-connected FSI pairs (Erisir et al., 1999; Lien and Jonas, 2003). TEA induced a significant increase in both AP width (ctrl: 0.43 ± 0.04 ms, TEA: 0.8 ± 0.1 ms, *n* = 6 pairs, *p* < 0.05, paired *t*-test) and amplitude (ctrl: 57 ± 4 mV, TEA: 68 ± 4 mV, *p* < 0.05), and a significant decrease in AHP amplitude (ctrl: 16 ± 2 mV, TEA: 8 ± 1 mV, *p* < 0.01). These changes were reflected in the postsynaptic spikelet as a significant increase in the ratio between peak and trough amplitudes (ctrl: 0.8 ± 0.1, TEA: 3.5 ± 1.0, *p* < 0.01, paired *t*-test; Figures 8A,B). The CC did not change after TEA application (ctrl: 0.06 ± 0.01, TEA: 0.05 ± 0.01, *p* > 0.05 paired *t*-test). After blocking Kv3 channels, the spike inhibitory effect was reversed as FSI pairs mainly fired AP in synchrony (PMFR ctrl: −0.16 ± 0.04, TEA: 0.2 ± 0.08, *n* = 6, *p* < 0.05, paired *t*-test; Figures 8C,D). These data demonstrate that the relatively slow hyperpolarizing phase of spikelets, favored by GJ low-pass filtering properties at the expense of the early fast depolarizing phase, is responsible for a net V_m_ hyperpolarization in response to presynaptic AP and causes inhibition of postsynaptic firing in GJ-connected FSI pairs.

## Discussion

We investigated the role of GJ in shaping the firing activity of electrically connected FSI pairs recorded in mouse neostriatal slices. Whereas individual APs were either synchronized or inhibited at relatively low firing frequencies (2–20 Hz), a strong inhibition of AP firing prevailed during presynaptic burst activity at an intra-burst frequency of 25–60 Hz and postsynaptic firing (either tonic or burst-like) at less than 50 Hz. In pairs composed by two bursting FSIs, GJ often induced burst alternation. In parallel, GJ promoted a relatively slow coupling of membrane potential which allowed the entrainment of connected FSIs into a loosely coordinated firing activity. The magnitude of the AP inhibitory effect was proportional to the amplitude of Gc and was mediated by the hyperpolarizing phase of postsynaptic spikelets. Spike inhibition persisted in the presence of the GABA_A_-receptor antagonist gabazine, suggesting that, although they might partially contribute to the effect, fast GABAergic synapses are not necessarily required for the repetitive inhibition of firing activity.

Gap junctions have been shown to induce spike synchrony between cell pairs (including PV^+^-FSIs similar to the striatal ones described here) in several brain regions (Galarreta and Hestrin, 1999; Gibson et al., 1999; Mann-Metzer and Yarom, 1999; Tamas et al., 2000; Hormuzdi et al., 2001; Szabadics et al., 2001; Traub et al., 2001; Landisman et al., 2002; Bennett and Zukin, 2004; Connors and Long, 2004; Christie et al., 2005; Long et al., 2005; Curti et al., 2012; but see Sippy and Yuste, 2013). Indeed, the occurrence of one or more spikelets at near-threshold potentials may actually trigger AP firing rather than cause inhibition (Mann-Metzer and Yarom, 1999; Curti et al., 2012). Here, however, the fast depolarizing phase of spikelets was not sufficient to reach firing threshold in cells having a relatively low input resistance (~70 MΩ at resting V_m_) such as striatal FSIs. In addition, we did not detect a substantial change in the depolarizing phase of spikelets at near-threshold V_m_ as compared to more negative potentials, suggesting that voltage-dependent conductances did not critically contribute to boost the spikelet excitatory phase which may result in AP triggering (Mann-Metzer and Yarom, 1999; Curti et al., 2012). Conversely, the slower hyperpolarizing phase provided effective and reliable inhibition, in particular during repetitive presynaptic firing which promoted temporal summation of spikelet AHPs. Pharmacological reduction of the presynaptic AHP resulted in a reversal of the ratio between the spikelet depolarizing peak and hyperpolarizing trough, which was sufficient to cause an overall synchronous spike activity in coupled FSIs. Such effect supports the view that electrical synapses exert spike inhibition by means of a net hyperpolarizing effect associated with temporal summation of spike AHPs propagating through GJ, which is particularly efficient at frequencies above 25 Hz.

Computational studies have predicted anti-phased firing (often in a bi-stable equilibrium with synchronization) in simulated electrically coupled cell pairs or networks (Chow and Kopell, 2000; Lewis and Rinzel, 2003; Nomura et al., 2003; Pfeuty et al., 2003; Mancilla et al., 2007), particularly when spikelets displayed a prevalently hyperpolarizing waveform (Ostojic et al., 2009; Otsuka and Kawaguchi, 2013). In modeled striatal networks, GJ induced a shunting effect by which two FSIs respond to uncoordinated excitatory input with a reduction in their firing frequency (Hjorth et al., 2009). In the cerebellum, pairs of electrically connected Golgi cells exhibited tightly synchronous firing (Dugue et al., 2009; Vervaeke et al., 2010), which transiently became anti-synchronous after eliciting an excitatory input from the mossy fibers (Vervaeke et al., 2010). Conversely, striatal FSI pairs described here displayed distinct properties in that (1) although both positive and negative spike-to-spike correlations occurred at low firing frequencies, burst activity at frequencies >25 Hz was mainly inhibitory over postsynaptic firing (2) both GJ-mediated V_m_ coupling and spike inhibition could be induced in the absence of synaptic stimulation after blocking AMPA and GABA_A_ receptors, suggesting that they were exclusively mediated by the interplay between GJ and intrinsic membrane properties, and (3) in robustly coupled pairs spike inhibition persisted throughout all presynaptic firing trains and did not appear to be a transient phenomenon. A clear differential mechanism by which GJ promote spike inhibition in the striatum as opposed to other areas remains to be elucidated. In cortical FSIs recorded in the same slices used for striatal experiments, we could detect a similar V_m_ coupling but not the spike inhibition effect (our unpublished results). Further work will assess how cell-specific combinations of dendritic morphology, membrane input resistance, ion channel expression, and GJ localization may contribute to critical differences between electrical coupling in striatal FSIs and their counterparts in other brain areas.

The overall lack of inhibitory effects by GABAergic synapses recorded in low [Cl^−^]_*i*_ was surprising, especially considering the strong GABAergic inhibition exerted by FSIs onto postsynaptic MSNs in slices (Koos and Tepper, 1999; our unpublished observation). Unitary IPSCs recorded in voltage-clamp mode, when present, were usually small in amplitude (< −40 pA at −70 mV). Although most of our recordings were conducted in whole-cell configuration, a few experiments using perforated-patch revealed a similar reversal potential for GABAergic currents (approximately −60 mV), suggesting that the relatively weak IPSCs were not due to an artificially small driving force imposed by our recording conditions. One cannot exclude that the simultaneous activation of multiple presynaptic FSIs may exert some significant compound inhibition over AP firing; in any case, given the observation that inhibition occurred even after blocking GABA_A_ receptors with gabazine, we suggest that in adult slices GABAergic synapses may provide inhibitory control of dendritic excitability and integrating properties rather than a direct block of AP firing. The relatively fast kinetics of IPSCs recorded in this study suggest that such integration likely occurs across proximal dendritic segments (Kita et al., 1990).

Results shown in Figure 3B suggest that GJ, by means of subthreshold V_m_ coupling, may assist concurrent excitatory inputs to reach firing threshold in neighboring FSIs and promote coherent firing in a local network of connected interneurons. This form of coincidence detection may occur in response to a transient increase in input correlation (Hjorth et al., 2009), e.g., during synchronous transitions to up-states (Blackwell et al., 2003; Gruber et al., 2009; Schulz et al., 2011) or in correspondence of activity strongly correlated to cortical rhythms (Sharott et al., 2012). In contrast, fast AP inhibition provides a reliable and temporally precise uncoupling in FSI firing which in turn may differentially distribute AP-dependent GABAergic feed-forward inhibition onto postsynaptic MSNs. This effect may be scaled up to relatively large local striatal FSI networks, which have been shown to be generally desynchronized using realistic simulations (Hjorth et al., 2009; Humphries et al., 2009) and also to maintain a globally desynchronized state in the network formed by striatal principal cells (Humphries et al., 2009). *In vivo*, AP inhibition may account for the overall lack of coherent firing activity (Berke, 2008) and the sharp negative cross-correlation peak (Lansink et al., 2010) detected in striatal FSIs of freely moving rats. On the other hand, membrane potential coupling may support transient co-activation of FSIs occurring in association with engagement in a left-right choice task during operant behavior (Gage et al., 2010). A direct detection of V_m_ dynamics that regulate FSI excitability would greatly help identify the action of GJ during specific tasks *in vivo*—although this represents a difficult task to pursue given the scarcity of striatal FSIs, which should be recorded “blindly” using intracellular electrodes (Gruber et al., 2009; Schulz et al., 2011).

Based on model simulations in which suprathreshold DC injection resulted in synchronization of burst-like firing in FSI pairs, Klaus et al. (2011) recently proposed that lack of synchronized firing in extracellularly recorded striatal FSIs *in vivo* suggests that burst firing activity in these cells is not driven by a sustained V_m_ depolarization—such as that induced by neuromodulators like dopamine (Bracci et al., 2002), serotonin (Blomeley et al., 2009), and acetylcholine (Koos and Tepper, 2002) or following activation of mGluR1 (Bonsi et al., 2007)—but is rather triggered by a rapid fluctuation of glutamatergic EPSPs. Although this hypothesis cannot be ruled out, our results alternatively suggest that burst firing trains induced by steady current injection *do* directly inhibit firing activity in an electrically connected cell. Thus, lack of synchronization *in vivo* does not necessarily imply that firing is driven solely by phasic glutamatergic input and not by a relatively steady, depolarized up-state.

The integrative properties of electrical synapses may be altered under pathological conditions such as dopamine depletion—a hallmark of Parkinson's disease—where striatal cell assemblies become entrained into excessively dominant states of synchronization (Jaidar et al., 2010). In fact, morphological and electrophysiological changes occurring in animal models of striatum-related disorders such as Parkinson's disease (Gittis et al., 2011) and dystonia (Gernert et al., 2000), as well as in human Tourette syndrome (Kalanithi et al., 2005), underlie the critical importance of FSIs in controlling the functioning of striatal networks. FSI microcircuits therefore represent a fundamental target for investigating therapeutic strategies against highly incident and debilitating pathologies.

In conclusion, electrical synapses between identified FSIs in mouse dorsal striatum induced a marked, frequency-dependent inhibition of AP firing together with a loose entrainment into coherent firing through V_m_ coupling. Firing inhibition was (1) imposed by the spike AHP propagating through GJ, (2) independent of the presence of GABAergic synapses, and (3) directly proportional to the strength of the GJ coupling. The versatile integrative functions of electrical synapses attribute FSIs a complex role in modulating the activity of local striatal networks.

## Author contributions

Giovanni Russo performed electrophysiological recordings and analyzed data; Thierry R. Nieus designed the spike train analysis, modeled the randomized EPSCs and analyzed data; Silvia Maggi analyzed data; Stefano Taverna designed experiments, analyzed data and wrote the manuscript.

### Conflict of interest statement

The authors declare that the research was conducted in the absence of any commercial or financial relationships that could be construed as a potential conflict of interest.

## Supplementary material

Figure S1 **Synchronizing effect of depolarizing GABA. (A)** I-V plot of GABAergic currents recorded using a high intracellular Cl^−^ solution (114 mM). The dotted line is a linear fit of data points extrapolated to the intersection with the x-axis (estimated *E*_*Cl*_ = −4 mV). Inset, individual depolarizing postsynaptic potential (dPSP, black trace) evoked at a V_m_ of −52 mV by a presynaptic AP (blue trace). **(B)** Co-activation of AP firing during short pulses of supra-threshold current injected in FSI_2_. This pair was connected via GABAergic synapses only. Inset: voltage response to a hyperpolarizing current injection in FSI_2_ evoked no V_m_ coupling in FSI_1_, i.e., the two cells were not connected through electrical synapses. Differently from pairs connected via GJ, here AP synchronization was induced by GABAergic depolarizing PSPs. **(C)** During firing activity evoked by DC injection (300–400 pA), individual spikes were tightly synchronized (top). Firing activity became mostly asynchronous after blocking GABA_A_-Rs via bath application of 10 μ M gabazine. **(D)** Cross-correlograms relative to traces in **(C)**. A significant peak at ~3 ms was visible in control conditions but not after application of gabazine.
