# Supplementary figures and images for: Dynamics of action potential firing in electrically connected striatal fast-spiking interneurons
Source: Front Cell Neurosci. 2013 Nov 14;7:209. doi: 10.3389/fncel.2013.00209 (PMC3827583; doi:10.3389/fncel.2013.00209)

# Supplementary Figure S1

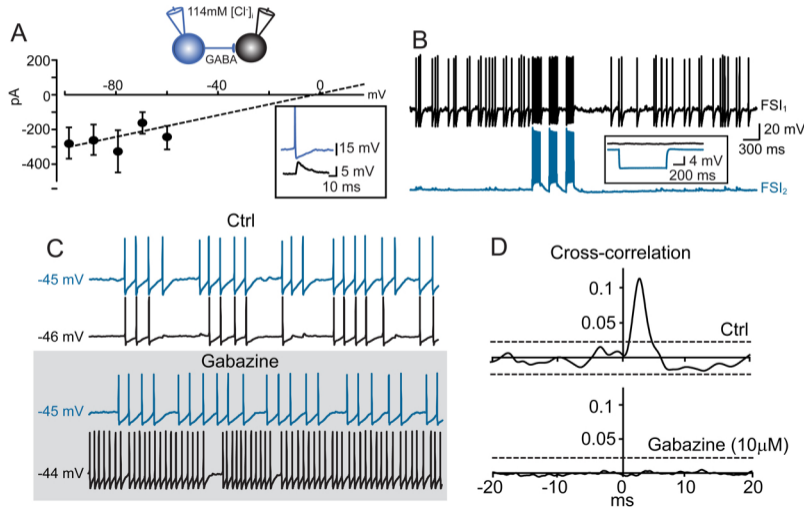

Supplement: Figure S1 — Synchronizing effect of depolarizing GABA. (A) I-V plot of GABAergic currents recorded using a high intracellular Cl− solution (114 mM). The dotted line is a linear fit of data points extrapolated to the intersection with the x-axis (estimated ECl = −4 mV). Inset, individual depolarizing postsynaptic potential (dPSP, black trace) evoked at a Vm of −52 mV by a presynaptic AP (blue trace). (B) Co-activation of AP firing during short pulses of supra-threshold current injected in FSI2. This pair was connected via GABAergic synapses only. Inset: voltage response to a hyperpolarizing current injection in FSI2 evoked no Vm coupling in FSI1, i.e., the two cells were not connected through electrical synapses. Differently from pairs connected via GJ, here AP synchronization was induced by GABAergic depolarizing PSPs. (C) During firing activity evoked by DC injection (300–400 pA), individual spikes were tightly synchronized (top). Firing activity became mostly asynchronous after blocking GABAA-Rs via bath application of 10 μ M gabazine. (D) Cross-correlograms relative to traces in (C). A significant peak at ~3 ms was visible in control conditions but not after application of gabazine. [file Presentation1.PDF]
